# Associations of *LBX1* gene and adolescent idiopathic scoliosis susceptibility: a meta-analysis based on 34,626 subjects

**DOI:** 10.1186/s12891-016-1139-z

**Published:** 2016-07-22

**Authors:** Yaqin Cao, Jikang Min, Qianghua Zhang, Heng Li, Haidong Li

**Affiliations:** Department of Orthopaedics, the First People’s Hospital of Huzhou, 158 Guangchanghou Road, Huzhou, Zhejiang Province 313000 China

**Keywords:** LBX1 gene, Adolescent idiopathic scoliosis, Gene polymorphism, Meta-analysis

## Abstract

**Background:**

The results of studies investigating the association between the ladybird homeobox 1 (*LBX1*) gene polymorphisms and the risk of adolescent idiopathic scoliosis (AIS) are not all the same. As such, we performed a meta-analysis to estimate the association between *LBX1* gene polymorphisms and AIS susceptibility.

**Methods:**

Relevant studies published before 15 November 2015 were identified by searching PubMed, EMBASE, ISI web of knowledge, EBSCO, CNKI and CBM. The strength of relationship was assessed by using odds ratios (ORs) and 95 % confidence interval (CI).

**Results:**

A total number of eight case-control studies including 10,088 cases and 24,538 controls were identified. The results showed that T allele of rs111090870 increased AIS susceptibility in Asians (T vs. C, OR = 1.22, 95 % CI: 1.16–1.29, *P* < 0.001), Caucasians (T vs. C, OR = 1.17, 95 % CI: 1.14–1.21, *P* < 0.001) and in female (T vs. C, OR = 1.21, 95 % CI: 1.17–1.25, *P* < 0.001). The G allele of rs678741 decreased AIS risk in female (G vs. A, OR = 0.83, 95 % CI: 0.81–0.85, *P* < 0.001), and the G allele of the rs625039 increased AIS susceptibility in Asians (G vs. A, OR = 1.14, 95 % CI: 1.11–1.17, *P* < 0.001).

**Conclusions:**

Our meta-analysis provides evidence that rs111090870, rs678741 and rs625039 polymorphisms near *LBX1* gene are associated with AIS susceptibility in some populations. However, our findings are based on only a limited number of studies.

**Electronic supplementary material:**

The online version of this article (doi:10.1186/s12891-016-1139-z) contains supplementary material, which is available to authorized users.

## Background

Adolescent idiopathic scoliosis (AIS) is a medical condition which affects 1–4 % of children in the at-risk population of those aged 10–16 years, using a cut-off point of 10° Cobb angle or more [1, 2]. An X-ray of an AIS patient is showing in Additional file 1: Figure S1. Without a known cause, AIS has been simply defined as a structural lateral curvature. However, the deformity is three-dimensional, which entails the coronal, sagittal and transverse planes of the spine [3, 4]. AIS leads to significant functional disabilities, especially pulmonary impairment [5, 6]. It also causes pain and cosmetic problems [7, 8]. The cause of AIS is complex and possible ethiology pathogeneses include genetic factors, hormones and metabolic dysfunction, abnormal growth, and environmental and life style factors [9–12]. Of all these factors, genetic factors are widely-accepted and well-documented [13–15]. Many genes involving in the initiation and evolution of AIS have been identified as its susceptible genes, such as Melatonin Receptor 1B (*MTNR1B*), ladybird homeobox 1 (*LBX1*), tryptophan hydroxylase 1 (*TPH1*), arylalkylamine N-acetyltransferase (*AA-NAT*) and Basonuclin 2 (*BNC2*) [1, 16–18], and among them, the *LBX1* gene is widely investigated.

The *LBX1* gene locates on chromosome 10q24.31, and it is a hemeobox transcription factor [19, 20]. It takes part in spinal cord differentiation and patterning, and somatosensory signal transduction. Therefore, *LBX1* is a strong biological candidate gene for AIS [21, 22]. *LBX1* encodes ladybird homeobox 1, orthologous to *Drosophila ladybird-late*, which plays a key role in regulation of muscle precursor cell migration and highly functional in central nervous system [19]. Large-scale genome-wide (GWAS) association studies conducted in Japanese and Chinese have tried to identify single nucleotide polymorphisms (SNPs) in relation to AIS risk [19, 23]. In 2015, several new studies which concerned on *LBX1* SNPs polymorphisms and AIS susceptibility have been published in Caucasian subjects [24, 25]. The results of these studies showed that several allelic polymorphisms near *LBX1* gene may act as potential susceptible factors for AIS, such as rs111090870, rs11598564 and rs625039. However, the results are not all the same and with limited statistical power among these studies. In order to overcome the limitation of single studies, we performed this meta-analysis containing one widely studied locus (rs111090870) and three less studied loci (rs678741, rs11598564 and rs625039), to provide a more comprehensive and precise estimation of *LBX1* gene and AIS susceptibility.

## Methods

### Data sources

Six databases were electronically searched, including PubMed, EMBASE, ISI web of knowledge, EBSCO, China National Knowledge Infrastructure (CNKI), and Chinese Biological and Medical Database (CBM), to retrieve studies analyzing the association between AIS susceptibility and *LBX1* gene polymorphisms until 1 November 2015. Searching terms were: “adolescent idiopathic scoliosis” or “AIS”, in combination with “LBX1” or “ladybird homeobox 1” or “HPX6” or “homeobox”, and in combination with: “polymorphism” or “variant” or “genotype” or “allele”. We also checked the reference lists of all included studies to make sure no study was missed.

### Inclusion criteria

We first performed initial screening of titles and abstract. A second round screening was based on full-text reviews. Studies were considered eligible if they met the following criteria: (1) It was a case-control study in design; (2) It evaluated the *LBX1* gene polymorphisms and AIS susceptibility; (3) AIS was diagnosed on the basis of clinical and radiologic examinations; (4) Individual genotype frequencies or allele frequencies in cases and controls were available.

### Exclusion criteria

Researches were excluded if they met any one of the following criteria: (1) Data came from reviews or abstracts; (2) Genotype and allele frequencies were both unavailable; (3) Repeatedly published literature.

### Data extraction and quality assessment

Two reviewers independently searched and selected literature, and then, extracted relevant data according to a data extraction form. Disagreements were solved by discussion until consensus was made. The extracted data included: the first author, year of publication, country of origin, ethnicity of the study population, genotyping method, source of control, sample size, the genotype and allele frequencies of the *LBX1* gene polymorphisms, and information of Hardy-Weinberg equilibrium (HWE) in control group.

Quality assessment was conducted for each article according to a quality evaluation form base on Critical Appraisal Skills Programme (CASP) for case-control study, which containing eleven questions associated with information provided in single studies [26]. Each question has three degrees, “yes” (scored 2), “can’t tell” (scored 1), or “no” (scored 0). After evaluating each question, a total score from 0 to 22 was given to each article. Studies included in this meta-analysis were divided into 3 grades: Grade A (high quality, scored 15–22), Grade B (medium quality, scored 8–14), Grade C (inferior quality, scored 0–7).

### Statistical analysis

Data analysis was conducted using STATA 11.0 software (Stata Statistical software, College Station, TX, USA, www.stata.com). Odds ratio (OR) and its corresponding 95 % confidence intervals (95 % CI) were used to evaluate the strength of association between *LBX1* gene polymorphisms and AIS susceptibility. Heterogeneity among included studies was tested using chi-square-based Q test and I^2^ test. *P*_*het*_ < 0.05 and *I*^*2*^ > 50 % were considered as statistically significant for heterogeneity. The Mantel-Haenszel method was used for fix effect model if no heterogeneity was found. Otherwise, the DerSimonian-Laird random effect model was used. Fix effect model considers that across all studies, the genetic factors have similar effects on genetic disorder susceptibility and the observed differences among studies are cause just by chance [27]. Random effect model considers that different studies may have substantial diversity, and it calculates within- as well as between- study difference [28]. Five comparison genetic models were used to assess the association between *LBX1* gene polymorphisms and AIS susceptibility. For instance, for rs1190870 polymorphism, T allele, we assessed the dominant model (TT + CT vs. CC), the recessive model (TT vs. TC + CC), the allele contrast genetic model (T vs. C), the heterozygote comparison (CT vs. CC), and the homozygote comparison (TT vs. CC). HWE was tested for included studies if no relevant information was provided in original research. Sensitivity analyses were conducted by omitting individual studies sequentially. Moreover, we performed subgroup analysis stratified by ethnicity and gender. Publication bias was quantitatively assessed by Egger’s linear regression test [29] and visual inspection of Begg’s funnel plots.

## Results

### Literature search

We initially identified 86 potentially relevant studies from six databases searched. Firstly, we eliminate duplications, not case-control studies or irrelevant to *LBX1* polymorphisms. After this procedure, ten studies were retained. Then, we read the full tests of these articles, and we finally identified 8 case control studies eligible for meta-analysis [1, 19, 23, 24, 30–33], including 10,088 cases and 24,538 controls. One study [34] was excluded for it reported the same datasets as Takahashi et al., but was less detailed. Another study was excluded for genotype and allele frequencies were both unavailable in original research [35]. A flow chart of article selection process is described in Additional file 1: Figure S2.

### Studies characteristics

Table 1 presents the main characteristics of included studies and genotype frequencies of included studies can be found in Additional file 1: Table S1. Of the eight studies, seven [1, 19, 23, 24, 30, 31, 33] were published in English and one [32] was a Chinese doctoral dissertation. There were six studies carried out among Asians [19, 23, 30–33], and two among Caucasians [1, 24]. All studies included were case-control studies in design, and all patients with AIS fulfilled the diagnosis of scoliosis. The number ranged from 94 to 4317 for cases, and 182 to 9823 for controls. Controls were mainly normal healthy populations randomly recruited from general population, who were matched with cases in ethnicity, gender and age. Seven studies containing a total of 13 datasets tested the rs111090870 polymorphism and AIS susceptibility, including seven datasets for female, five for male and one mix gender dataset. Two studies analyzed rs678741, including five datasets for female. Two studies tested rs11598564, and they contained three datasets for female and two for male. Three studies tested rs625039 polymorphism, including three datasets for female, two for male and one mix gender dataset. In quality assessment, all studies included were categorized as grade A, with scores from 15 to 20 (Table 1). Only in one dataset, the genotype distributions in control groups were deviated from HWE.

**Table 1:** Characteristics of the datasets included in meta-analysis on association between *LBX1* polymorphisms and AIS

First author	Year	Country	Ethnicity	Case number (all cases, male/female)	Control number (all controls, male/female)	Cobb angles degrees of the included patients	Genotyping method	Quality grade	SNP tested and included in meta-analysis
Chettier et al.	2015	USA	Caucasian	620 female cases	1287 female controls	More than 10 degrees	Affymetrix HuSNP 6.0 Microarray	A (scored 15)	rs111090870, rs678741
Fan et al.	2012	China	Asian	300, 52/248	788, 299/489	More than 35 degrees	PCR-based invader assay	A (scored 18)	rs111090870
Gao et al.	2013	China	Asian	513, 66/447	440, 151/289	25.57 ± 14.10 degrees	PCR-MassArray assay	A (scored 16)	rs111090870, rs11598564, rs625039
Grauers et al.	2015	Sweden and Denmark	Caucasian	1739, 241/1498	1812, 0/1812	38.8 ± 17.5 degrees	MassArray assay	A (scored 15)	rs111090870
Jiang et al.	2013	China	Asian	949, 129/820	976, 314/662	More than 20 degrees	PCR-based invader assay	A (scored 15)	rs111090870
Liu	2015	China	Asian	180, 29/151	182, 30/152	NA	PCR-MassArray assay	A (scored 17)	rs111090870, rs625039
Takahashi et al.	2012	Japan	Asian	1453, 94/1359	13127, 1849/11278	More than15 degrees	I PCR-based invader assay, llumina Human610 and HumanHap550v3 microarrays	A (scored 20)	rs111090870, rs11598564, rs625039
Zhu et al.	2015	China	Asian	4317 female cases	6016 female controls	37.2 ± 9.4 degrees	Affymetrix Genome-wide Human SNP array 6.0	A (scored 15)	rs678741

*AIS* adolescent idiopathic scoliosis; *GWAS* genome-wide association; *USA* United States of America; *PCR* polymorphism chain reaction; *SNP* single nucleotide polymorphism, NA not applicable

### Quantitative data analysis

#### rs111090870 polymorphism and AIS susceptibility

**Fig. 1:**
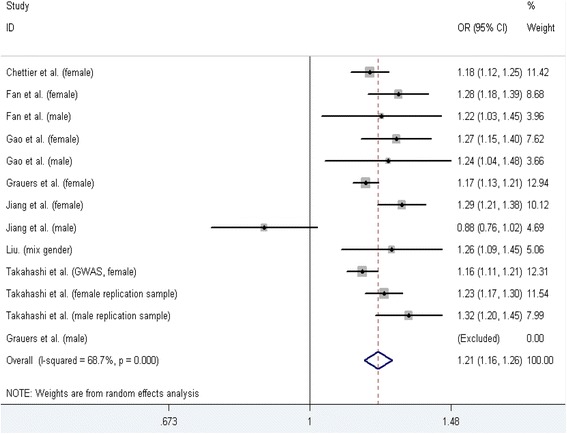
Meta-analysis forest plot of the association between rs11109070 polymorphism and AIS risk (allele contrast genetic model, T vs. C)

Seven case-control studies [1, 19, 23, 24, 30–32] containing 13 datasets on relationship between rs111090870 polymorphism and AIS susceptibility were identified, including 5754 cases and 18,628 controls. The results of five genetic models testing rs111090870 polymorphism and AIS susceptibility were showed in Table 2. A significant increase in AIS susceptibility was found in all of five genetic models. In the subgroup analysis stratified by gender, significant increasing AIS susceptibility was found for female in the dominant model (TT + TC vs. CC: OR = 1.13, 95 % CI: 1.09–1.16, *P* < 0.001) and allele contrast genetic model (T vs. C: OR = 1.21, 95 % CI: 1.17–1.25, *P* < 0.001). In mix gender subgroup, allele contrast genetic model also showed a significant increase (T vs. C: OR = 1.26, 95 % CI: 1.09–1.45, *P* = 0.001), but no significant association between rs111090870 and AIS risk was found in male subgroup. In subgroup analyses stratified by ethnicity, significant increasing AIS susceptibility was found for Asians and Caucasians in both genetic models (Table 3). Figure 1 shows the forest plot of allele contrast genetic model testing the association between rs11109070 polymorphism and AIS risk.

**Table 2:** Summary of different genetic model comparison results

SNP	Genetic model	OR (95 % CI)	*Z*	*P* value	*I* ^*2*^%	*P*_*het*_	Effect model	Egger’s test	
								*t* value	*P* value
rs111090870	TT + TC vs. CC	1.12 (1.09–1.15)	7.66	0.000	68.8	<0.001	R	0.42	0.684
	TT vs. TC + CC	1.42 (1.36–1.49)	15.49	0.000	42.6	0.058	F	0.92	0.378
	TT vs. CC	1.36 (1.27–1.45)	9.07	0.000	73.2	<0.001	R	0.98	0.349
	TC vs. CC	1.13 (1.09–1.18)	5.92	0.000	56.3	0.009	R	−0.07	0.949
	T vs. C	1.21 (1.16–1.26)	9.52	0.000	68.7	<0.001	R	0.55	0.597
rs678741	GG + GA vs. AA	0.79 (0.77–9.82)	16.79	0.000	0.0	0.998	F	0.85	0.458
	GG vs. GA + AA	0.69 (0.64–0.74)	10.98	0.000	0.0	0.880	F	−0.82	0.470
	GG vs. AA	0.68 (0.65–0.72)	13.22	0.000	0.0	0.517	F	−1.09	0.356
	GA vs. AA	0.88 (0.85–0.91)	8.12	0.000	0.0	0.904	F	−0.73	0.519
	G vs. A	0.83 (0.81–0.85)	13.75	0.000	0.0	0.743	F	−0.69	0.542
rs11598564	GG + GA vs. AA	1.13 (1.10–1.16)	7.98	0.000	0.0	0.649	F	0.32	0.769
	GG vs. GA + AA	1.09 (0.84–1.43)	0.64	0.519	88.8	<0.001	R	0.29	0.726
	GG vs. AA	1.12 (1.33–1.52)	10.12	0.000	0.0	0.761	F	−0.71	0.526
	GA vs. AA	1.13 (1.08–1.18)	5.34	0.000	0.0	0.739	F	1.02	0.384
	G vs. A	1.21 (1.16–1.25)	10.03	0.000	22.2	0.273	F	0.66	0.557
rs625039	GG + GA vs. AA	1.07 (1.05–1.09)	7.45	0.000	0.0	0.648	F	0.20	0.850
	GG vs. GA + AA	1.30 (1.23–1.37)	9.05	0.000	0.0	0.509	F	1.18	0.303
	GG vs. AA	1.17 (1.13–1.21)	9.48	0.000	0.0	0.651	F	0.66	0.545
	GA vs. AA	1.09 (1.05–1.12)	4.66	0.000	0.0	0.573	F	−0.35	0.747
	G vs. A	1.14 (1.11–1.17)	10.14	0.000	0.0	0.584	F	1.10	0.333

*SNP* single nucleotide polymorphism; *OR* odds ratio; *CI* confidence interval; *F* fix-effect model; *R* random-effect model; *P*_*het*_ *P* value for heterogeneity

*P* < 0.05 stands for statistical significance

**Table 3:** Results of subgroup analyses

SNP	Comparison	Number of datasets	Dominant genetic model		Allele contrast	
			OR (95 % CI)	*P* value	OR (95 % CI)	*P* value
rs111090870	Gender					
	Female	7	1.13 (1.09–1.16)	<0.001	1.21 (1.17–1.25)	0.000
	Male	5	1.08 (0.93–1.25)	0.319	1.15 (0.95–1.40)	0.015
	Mix gender	1	1.10 (0.99–1.23)	0.087	1.26 (1.09–1.45)	0.001
	Ethnicity					
	Asian	10	1.13 (1.09–1.17)	<0.001	1.22 (1.16–1.29)	0.000
	Caucasian	3	1.09 (1.08–1.22)	<0.001	1.17 (1.14–1.21)	0.000
rs678741	Ethnicity					
	Asian	4	0.79 (0.77–0.82)	<0.001	0.83 (0.81–0.86)	0.000
	Caucasian	1	0.80 (0.74–0.86)	<0.001	0.79 (0.73–0.86)	0.000
rs11598564	Gender					
	Female	3	1.14 (1.09–1.16)	<0.001	1.20 (1.15–1.25)	0.000
	Male	2	1.16 (1.08–1.23)	<0.001	1.27 (1.17–1.39)	0.000
rs625039	Gender					
	Female	3	1.07 (1.05–1.09)	<0.001	1.13 (1.10–1.17)	0.000
	Male	2	1.07 (1.02–1.13)	0.010	1.20 (1.12–1.29)	0.000
	Mix gender	1	1.05 (0.96–1.14)	0.256	1.14 (1.02–1.28)	0.025

#### rs678741 polymorphism and AIS susceptibility

**Fig. 2:**
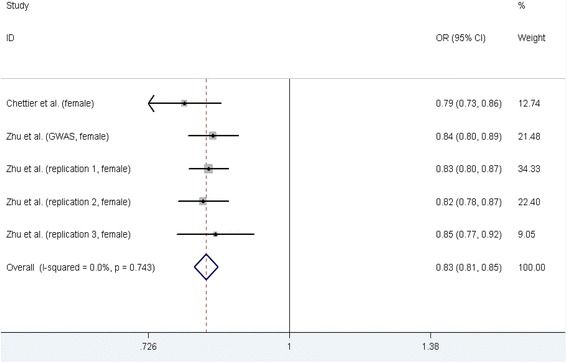
Meta-analysis forest plot of the association between rs678741 polymorphism and AIS risk (allele contrast genetic model, G vs. A)

Two case-control studies [1, 33] containing five datasets on relationship between rs678741 polymorphism and AIS susceptibility were identified, including 4937 cases and 7303 controls, and the five datasets all contained female participants. The results of five genetic models testing rs678741 polymorphism and AIS susceptibility were showed in Table 2. A significant decrease in AIS susceptibility was found in all of five genetic models. In the subgroup analysis stratified by ethnicity, significant decreasing AIS susceptibility was found for both ethnicities in dominant and allele contrast genetic models (Table 3). Figure 2 shows the forest plot of allele contrast genetic model testing the association between rs678741 polymorphism and AIS risk.

#### rs11598564 polymorphism and AIS susceptibility

**Fig. 3:**
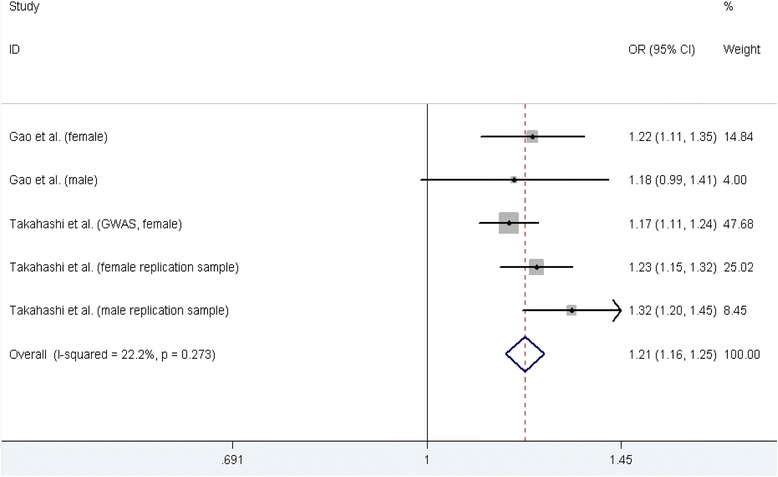
Meta-analysis forest plot of the association between rs11598546 polymorphism and AIS risk (allele contrast genetic model, G vs. A)

Two case-control studies [19, 30] containing five datasets on relationship between rs11598564 polymorphism and AIS susceptibility were identified, including 1966 cases and 13,585 controls and the five databases all contained Asian participants. The results of five genetic models testing rs11598564 polymorphism and AIS susceptibility were showed in Table 2. A significant increase in AIS susceptibility was found in all of five models except for recessive genetic model (GG vs. GA + AA, OR = 1.09, 95 % CI: 0.84–1.43, *P* = 0.519). In the subgroup analysis stratified by gender, significant increasing AIS susceptibility was found for both genders in the dominant model and allele contrast genetic model (Table 3). Figure 3 shows the forest plot of allele contrast genetic model testing the association between rs11598564 polymorphism and AIS risk.

#### rs625039 polymorphism and AIS susceptibility

**Fig. 4:**
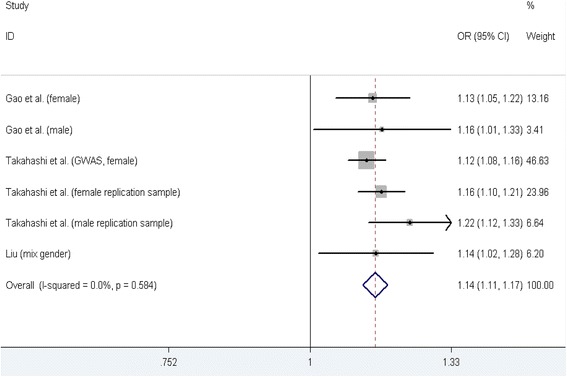
Meta-analysis forest plot of the association between rs625039 polymorphism and AIS risk (allele contrast genetic model, G vs. A)

Three case-control studies [19, 30, 32] containing six datasets on relationship between rs678741 polymorphism and AIS susceptibility were identified, including 1, 646 cases and 13,749 controls, and the six datasets all contained Asians participants. The results of five genetic models testing rs625039 polymorphism and AIS susceptibility were showed in Table 2. A significant increase in AIS susceptibility was found in all of five genetic models. In the subgroup analysis stratified by gender, significant increasing AIS susceptibility was found for both genders allele contrast genetic models (Table 3). Figure 4 shows the forest plot of allele contrast genetic model testing the association between rs625039 polymorphism and AIS risk.

#### Sensitivity analysis and publication bias

Sensitivity analyses were conducted by omitting each dataset sequentially. For four SNP polymorphisms, the result did not change under any genetic model. For rs11090870, when we omitted the male dataset reported by Jiang et al. [31], the indicators for heterogeneity was reduced under dominant, heterozygote, homozygote and allele contrast genetic models. For rs11598564, when we reduce the female dataset reported by Gao et al. [30], the indicators for heterogeneity was reduced under recessive genetic model. Sensitivity analysis suggested that the results for *LBX1* gene polymorphisms and AIS susceptibility were stable and statistically robust.

Visual inspection of Begg’s funnel plots did not identify substantial asymmetry for all SNPs under any genetic model (Additional file 1: Figure S3-S6). The Egger’s linear regression test also indicated no evidence of publication bias in studies testing *LBX1* gene polymorphisms and AIS susceptibility (*P* > 0.05 for all models tested) (Table 2).

## Discussion

Previous clinical and epidemiological studies found that about 27 % female offspring of female AIS patients would also suffer from AIS, but this phenomenon did not exist in male AIS patients, and that the pairwise concordance rate was higher in monozygotic twin pairs comparing with dizygotic twin pairs [36, 37]. So, the genetic factors play essential roles in pathogenesis of AIS. Preclinical medicine researches shows that, in *LBX1* mutant mice, the morphology and neuronal circuitry of the dorsal horn are aberrant and in mice lacking *LBX1*, cells types that arise in the ventral alar plate acquire more dorsal identities [22, 38]. Therefore, *LBX1* is essential for sensory pathways developments which relay touch and pain. In 1982, Pincott et al. found that the loss of proprioceptive innervation could result in asymmetrical weakness of the paraspinal muscles, and which may finally cause scoliosis [39]. As a result, it is quite reasonable to deduce that *LBX1* gene may be a target spot in pathogenesis of AIS.

Case-control study is a useful tool to detect gene and disease susceptibility. GWAS is a kind of case-control cohort study in essential, which is a powerful method to identify genetic association with diseases and has been applied in genetic predisposition studies increasingly. Therefore, GWAS is widely used in AIS susceptibility study, to identity risk genes for this most common and complex musculoskeletal system polygenic disease [35, 40, 41]. Fan et al. and Takahashi et al. [19, 23] first reported results of a GWAS analyzing the association between *LBX1* gene and AIS susceptibility in 2012 and their results showed statistical significant associations for rs111090870, rs11598564 and rs625039. From 2013 to 2015, several subsequent studies have conducted to investigate the *LBX1* polymorphisms in pathogenesis of AIS. However, the results are inconsistent, with some datasets found positive associations, and other find no relevance or even negative association. Several reasons, including different recruitment criteria, subjects’ characteristics, sample size, different ethnic population and gender, may lead to the inconsistency.

In order to avoid limitations of individual case-control or GWA study, we conducted this meta-analysis to pool the findings of rs111090870, rs678741 rs11598564 and rs625039 polymorphisms in all original studies. As we reported in result part, the eight included studies were of high quality judged by CASP standard. They clearly reported their participants choosing criteria, sample sizes, characteristics of cases and controls and genotyping methods. Moreover, except one dataset cannot be tested for HWE, only one dataset was deviated from HWE in the control group. Gender and ethnicity were matched in case and control group. The results showed that T allele of rs111090870 is significant associated with increased AIS susceptibility in Asians (T vs. C, OR = 1.22, 95 % CI: 1.16–1.29, *P* < 0.001), Caucasians (T vs. C, OR = 1.17, 95 % CI: 1.14–1.21, *P* < 0.001) and in female (T vs. C, OR = 1.21, 95 % CI: 1.17–1.25, *P* < 0.001). The G allele of rs678741 decreased AIS risk in female (G vs. A, OR = 0.83, 95 % CI: 0.81–0.85, *P* < 0.001), and the G allele of the rs625039 polymorphism may increase AIS susceptibility in Asians (G vs. A, OR = 1.14, 95 % CI: 1.11–1.17, *P* < 0.001). For the rs11598564 polymorphism, G allele may also increase AIS risk, but in recessive model, no statistical significant association was detected and the result was with heterogeneity. So, the result of rs11598564 should be interpreted with caution.

We should notice the heterogeneity existed in this meta-analysis. For the rs111598564 and rs625039 polymorphisms, no heterogeneity was found among studies. However, in the rs111090870 polymorphism, significant heterogeneity was found in all models except the recessive model, and for the rs11598564 polymorphism, heterogeneity was detected in recessive genetic models. For rs111090870 polymorphism, the heterogeneity detected in four genetic models was effectively decreased in sensitivity analysis when the male dataset in study by Jiang et al. [31] was omitted. In this study, the genotype distribution in control group was deviated from HWE. For rs11598564 polymorphism, when we stratified by gender, the heterogeneity existed in female group only, and when omitted individual dataset sequentially, we find female group in study by Gao et al. [30] contributed to the heterogeneity. Moreover, the removal of these datasets did not materially change the overall results of any genetic models. Therefore, deviation from HWE and different genders may contribute to overall heterogeneity of this meta-analysis.

Two previous meta-analyses [42, 43] have tried to analyze the association between rs111090870 polymorphism and AIS susceptibility in East Asians. For these two studies, all of the genetic models provided statistically significant comparison results. For instance, the ORs and their corresponding 95 % CI of the dominant models were 2.04 (2.27–3.03) and 2.02 (1.78–2.30) respectively. The results of our study were quite similar to theses to studies, but the effect sizes were lower than theirs, as the effect size of the dominant model in our study were 1.13 (1.09–1.17) for Asians and 1.12 (1.09–1.15) for overall population. Comparing with them, our meta-analysis has some important improvements. For rs111090870 polymorphism, some new published researches were included in our meta-analysis polymorphism, and through strict methodological process, we provided a more comprehensive view of included studies. The above mentioned meta-analysis only focus on east Asian participants, but our study also included Caucasian subjects and in subgroup studies, we stratified by ethnicity to test if there existed differences in variant ethnicities. For rs678741, rs11598564 and rs625039 polymorphisms, to the best of our knowledge, no published combined study has detected their association with AIS.

Several limitations of this study may affect the results. Firstly, only published English and Chinese studies were included in this meta-analysis and we only included published studies from six databases. Relevant studies in other languages and databases may have been missed. Secondly, in our meta-analysis, all datasets included in rs11598564 and rs625039 were based on Asian subjects. Additional researches in other ethnicities are needed to generalize our finding for rs11598564 and rs625039. For rs678741, all included datasets were based on female subjects, and other researches for males are needed. Because the same polymorphism may act differently in different ethnical backgrounds genders, results from rs678741 cannot extend to male and results from rs11598564, rs625039 cannot extend to Caucasians or other ethnicities. Thirdly, most included studies did not distinguish between the magnitude or phenotype of scoliosis and genotyping results. Therefore, we were unable to provide different pooled ORs according to different magnitude or phenotype of AIS. Finally, the possible ethiology pathogenesis of AIS is complex, but due to insufficiency of included studies, we did not detect the interactions between genetic factors and other factors. Considering that meta analysis is a kind of retrospective research and may easily be affected by methodological deficiencies of included studies, we developed a detailed protocol before conducting this analysis, to ensure the quality of our meta-analysis.

## Conclusion

From the combination results of currently included studies, our meta-analysis suggested that the T allele of rs111090870 polymorphism near *LBX1* gene is significant associated with increased AIS susceptibility in Asians, Caucasians and in female. The G allele of rs678741 may decrease AIS risk in female and the G allele of rs625039 polymorphism may increase AIS susceptibility in Asians. More studies with multiple ethnics and different genders are needed to generalize the results.

## Abbreviations

AA-NAT, arylalkylamine N-acetyltransferase; AIS, adolescent idiopathic scoliosis; BNC2, Basonuclin 2; CASP, Critical Appraisal Skills Programme; CBM, Chinese Biological and Medical Database; CI, confidence interval; CNKI, China National Knowledge Infrastructure; GWAS, Large-scale genome-wide; HWE, Hardy-Weinberg equilibrium; LBX1, ladybird homeobox 1; OR, odds ratios; PCR, Polymorphism chain reaction; SNP, single nucleotide polymorphism; TPH1, tryptophan hydroxylase 1

## Additional file

Additional file 1:
**Table S1.** Genotype and allele distribution of LBX1 polymorphisms in AIS cases and controls. **Figure S1.** X-ray of an AIS patient. **Figure S2.** Flow chart of data selection. **Figure S3.** Begg’s funnel plot for publication bias in the dominant model for rs111090870 polymorphism. **Figure S4.** Begg’s funnel plot for publication bias in the dominant model for rs678741 polymorphism. **Figure S5.** Begg’s funnel plot for publication bias in the dominant model for rs11598564 polymorphism. **Figure S6.** Begg’s funnel plot for publication bias in the dominant model for rs625039 polymorphism. (DOCX 144 kb)
