# Supplementary material for: Associations of LBX1 gene and adolescent idiopathic scoliosis susceptibility: a meta-analysis based on 34,626 subjects
Source: BMC Musculoskelet Disord. 2016 Jul 22;17:309. doi: 10.1186/s12891-016-1139-z (PMC4957912; doi:10.1186/s12891-016-1139-z)
Supplement: Additional file 1: — Table S1. Genotype and allele distribution of LBX1 polymorphisms in AIS cases and controls. Figure S1. X-ray of an AIS patient. Figure S2. Flow chart of data selection. Figure S3. Begg’s funnel plot for publication bias in the dominant model for rs111090870 polymorphism. Figure S4. Begg’s funnel plot for publication bias in the dominant model for rs678741 polymorphism. Figure S5. Begg’s funnel plot for publication bias in the dominant model for rs11598564 polymorphism. Figure S6. Begg’s funnel plot for publication bias in the dominant model for rs625039 polymorphism. (DOCX 144 kb) [file 12891_2016_1139_MOESM1_ESM.docx]

Table S1 Genotype and allele distribution of *LBX1* polymorphisms in AIS cases and controls.

| Locus | Study | Case | | | Control | | | Case | | Control | | Deviation from HWE |
| --- | --- | --- | --- | --- | --- | --- | --- | --- | --- | --- | --- | --- |
|  |  | TT | TC | CC | TT | TC | CC | T | C | T | C |  |
| rs111090870 | Chettier et al. (female)* | 262 | 282 | 76 | 389 | 637 | 261 | 806 | 434 | 1415 | 1159 | No |
|  | Fan et al. (female) | 114 | 109 | 25 | 136 | 246 | 106 | 337 | 159 | 518 | 458 | No |
|  | Fan et al. (male) | 19 | 27 | 6 | 80 | 147 | 73 | 65 | 39 | 307 | 293 | No |
|  | Gao et al. (female) | 171 | 213 | 63 | 74 | 134 | 81 | 555 | 339 | 282 | 296 | No |
|  | Gao et al. (male) | 29 | 23 | 14 | 40 | 69 | 42 | 81 | 51 | 149 | 153 | No |
|  | Grauers et al. (female)* | 713 | 641 | 144 | 631 | 877 | 305 | 2067 | 929 | 2138 | 1486 | No |
|  | Grauers et al. (male)* | 105 | 108 | 28 | 0 | 0 | 0 | 318 | 164 | 0 | 0 | NA |
|  | Jiang et al. (female) | 307 | 399 | 114 | 156 | 321 | 185 | 1013 | 627 | 633 | 691 | No |
|  | Jiang et al. (male) | 28 | 65 | 36 | 80 | 175 | 59 | 121 | 137 | 335 | 293 | Yes |
|  | Liu. (mix gender) | 63 | 84 | 33 | 34 | 101 | 47 | 210 | 150 | 169 | 195 | No |
|  | Takahashi et al. (GWAS, female) | 449 | 470 | 114 | 479 | 728 | 266 | 1368 | 698 | 1686 | 1260 | No |
|  | Takahashi et al. (female replication sample) | 152 | 148 | 26 | 3129 | 4809 | 1883 | 452 | 200 | 11067 | 8575 | No |
|  | Takahashi et al. (male replication sample) | 49 | 38 | 7 | 574 | 882 | 393 | 136 | 52 | 2030 | 1668 | No |
| rs678741 |  | GG | GA | AA | GG | GA | AA | G | A | G | A |  |
|  | Chettier et al. (female)* | 86 | 290 | 243 | 288 | 642 | 357 | 463 | 777 | 1218 | 1356 | No |
|  | Zhu et al. (GWAS, female)* | 219 | 498 | 283 | 464 | 741 | 296 | 937 | 1065 | 1668 | 1332 | No |
|  | Zhu et al. (replication 1, female)* | 395 | 950 | 571 | 594 | 992 | 414 | 1740 | 2092 | 2181 | 1821 | No |
|  | Zhu et al. (replication 2, female)* | 193 | 470 | 286 | 575 | 949 | 391 | 857 | 1043 | 2099 | 1731 | No |
|  | Zhu et al. (replication 3, female)* | 96 | 224 | 130 | 179 | 297 | 123 | 416 | 484 | 656 | 544 | No |
| rs11598564 |  | GG | GA | AA | GG | GA | AA | G | A | G | A |  |
|  | Gao et al. (female) | 159 | 219 | 69 | 71 | 142 | 76 | 537 | 357 | 284 | 294 | No |
|  | Gao et al. (male) | 26 | 27 | 13 | 44 | 65 | 42 | 79 | 53 | 153 | 149 | No |
|  | Takahashi et al. (GWAS, female) | 297 | 508 | 228 | 310 | 724 | 439 | 1102 | 964 | 1344 | 1602 | No |
|  | Takahashi et al. (female replication sample) | 107 | 156 | 63 | 2107 | 4837 | 2879 | 370 | 282 | 9051 | 10595 | No |
|  | Takahashi et al. (male replication sample) | 49 | 38 | 7 | 574 | 882 | 393 | 136 | 52 | 2030 | 1668 | No |
| rs625039 |  | GG | GA | AA | GG | GA | AA | G | A | G | A |  |
|  | Gao et al. (female) | 221 | 195 | 31 | 117 | 129 | 43 | 637 | 257 | 363 | 215 | No |
|  | Gao et al. (male) | 36 | 23 | 7 | 59 | 70 | 22 | 95 | 37 | 188 | 114 | No |
|  | Takahashi et al. (GWAS, female) | 533 | 424 | 76 | 600 | 695 | 178 | 1490 | 576 | 1895 | 1051 | No |
|  | Takahashi et al. (female replication sample) | 172 | 135 | 19 | 3947 | 4579 | 1279 | 479 | 173 | 12473 | 7137 | No |
|  | Takahashi et al. (male replication sample) | 56 | 31 | 7 | 726 | 848 | 275 | 143 | 45 | 2300 | 1398 | No |
|  | Liu (mix gender) | 79 | 78 | 23 | 58 | 93 | 31 | 236 | 124 | 209 | 155 | No |

GWAS, genome-wide association; NA, not applicable; HWE, Hardy-Weinberg equilibrium.

*Genotype frequencies in these datasets were not provided directly, and were calculated by allele frequencies.


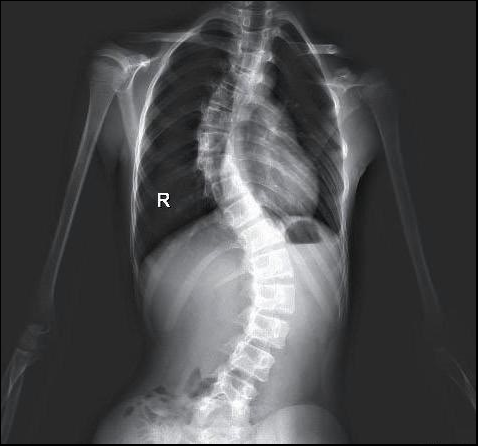


Figure S1 X-ray of an AIS patient.

Studies identified by searching PubMed, EMBASE, EBSCO, ISI web of knowledge, CNKI and CBM (n=86)

10 case-control studies including 13 datasets on association between *IL-13 rs20541* polymorphism and glioma susceptibility were included

10 articles relevant to *IL-13* polymorphism and glioma susceptibility were identified for full text reading

Studies for abstract reading after removing duplications (n=50)

40 studies were eliminated:

Not case-control studies (n=23)

Not relevant to *IL-13* gene *rs20541* polymorphism and glioma susceptibility (n=17)

2 studies were eliminated:

Reported the same datasets as another included study, but was less detailed (n=1)

Genotype and allele frequencies were both unavailable (n=1)

2 were excluded for:

Irrelevant to *rs20541* polymorphism (n=1)

Genotype frequencies and ORs for different genetic model were not provided (n=1)

8 case-control studies on association between *LBX1* polymorphisms and AIS susceptibility were included

Figure S2 Flow chart of data selection

Figure S3 Begg’s funnel plot for publication bias in the dominant model for rs111090870 polymorphism.

Figure S4 Begg’s funnel plot for publication bias in the dominant model for rs678741 polymorphism.

Figure S5 Begg’s funnel plot for publication bias in the dominant model for rs11598564 polymorphism.

Figure S6 Begg’s funnel plot for publication bias in the dominant model for rs625039 polymorphism.
